# Les dangers du SARS-COV-2 pour les écosystèmes aquatiques

**DOI:** 10.48327/mtsi.v2i1.2022.228

**Published:** 2022-03-29

**Authors:** Salma FERCHICHI, Naouel FATNASSI, Anissa DHAOUADI, Hédia ATTIA EL HILI

**Affiliations:** Centre national de veille zoosanitaire, 33 avenue Charles Nicolle, Tunis, Tunisie

**Keywords:** Une seule santé, Coronavirus, SARS-CoV-2, Contamination humain-animal, Écosystèmes aquatiques, Eaux usées, Synthèse bibliographique, One Health, Coronavirus, SARS-CoV-2, Human-animal contamination, Aquatic ecosystems, Sewage, Bibliographical synthesis

## Abstract

**Introduction:**

La pandémie actuelle de COVID-19 est due à l’apparition d’un nouveau coronavirus émergent SARS-CoV-2, famille des Coronaviridae et sous-famille des Orthocoronavirinae. Ce virus a été signalé pour la première fois en décembre 2019 en Chine. Bien que signalée par plusieurs pays chez plusieurs espèces animales, la COVID-19 est une maladie transmise principalement d’humain à humain. Par ailleurs, le SARS-CoV-2 ainsi que son ARN ont été détectés dans des excrétions corporelles autres que la salive, telles les urines et les matières fécales déversées dans les eaux usées.

**Revue bibliographique:**

Dans ce cadre, cet article présente une synthèse des revues bibliographiques sur le SARS-CoV-2 en milieu aquatique, tout en soulignant les généralités sur le SARS-CoV-2, les éventuelles sources de contaminations potentielles du SARS-CoV-2 dans les milieux hydriques, la viabilité du SARS-CoV-2 dans le milieu aquatique, les espèces réceptrices et les impacts du SARS-CoV-2 sur les écosystèmes aquatiques.

**Conclusion:**

Nous rassemblons les principales informations relatives au SARS-CoV-2 jugées importantes à retenir, et nous soulignons l’intérêt de poursuivre les recherches dans ce domaine pour évaluer les dangers que représente le SARS-CoV-2 pour les écosystèmes aquatiques.

## Abstract (en)

**Introduction:**

The current COVID-19 pandemic is due to a new emerging coronavirus SARS-CoV-2, belonging to the Coronaviridae family and to the Orthocoronavirinae subfamily. This virus was first reported in December 2019 in China. Although reported by several countries in several animal species, COVID-19 is a disease transmitted from human to human. Moreover, SARS-CoV-2 virus and its RNA were detected in body excretions besides saliva, such as urine and fecal matter discharged into sewage.

**Bibliographic review:**

Within this framework, this article aims to synthesize the bibliographical reviews on SARS-CoV-2 in aquatic environment. It will underline the generalities on SARS-CoV-2, the possible sources of potential contaminations of SARS-CoV-2 in water environment, the viability of SARS-CoV-2 in aquatic environment, the receptive species and the impacts of SARS-CoV-2 on the aquatic ecosystems.

**Conclusion:**

We compile key information about SARS-CoV-2 that are considered important to remember and highlight the importance of further research in this area in order to assess the hazards of SARS-CoV-2 on aquatic ecosystems.

## Introduction

Depuis décembre 2019, un nouveau Coronavirus émergent appelé SARS-CoV-2 et responsable de la maladie COVID-19 est apparu dans la ville de Wuhan, province de Hubei en Chine et s’est propagé très rapidement dans presque tous les pays du monde. Cette pandémie a généré des répercussions considérables d’ordre aussi bien sanitaire que socio-économique.

Il est actuellement connu que la principale transmission de la COVID-19 est interhumaine. Le virus en cause se transmet par voie aérienne notamment via des aérosols générés par la toux ou les éternuements, voire les postillons émis en parlant. Selon l’OMS, les personnes peuvent également être infectées en touchant des surfaces contaminées par le virus lequel est ensuite porté par les mains jusqu’aux yeux, au nez ou à la bouche avant qu’elles ne soient lavées [57]. En ce qui concerne les voies aériennes, le SARS-CoV-2 a été retrouvé dans celles des patients symptomatiques, mais aussi asymptomatiques.

Une étude faite par Marks et al en 2021 a démontré que l’expression des symptômes dépend de la charge virale en SARS-CoV-2 initiale. En effet, sur les 421 cas contacts ayant eu une PCR positive à l’inclusion et qui étaient asymptomatiques, 43 % ont développé une forme symptomatique de COVID-19 au cours de la période de suivi. Cela concernait davantage (66 %) ceux qui avaient une charge virale élevée à l’inclusion (> 1,10^10^ copies/ml) que ceux dont la charge virale était plus basse (seulement 38 % de ceux qui avaient une charge de 7 copies/ml) [50].

Néanmoins, une transmission oro-fécale est possible puisque plusieurs études ont décrit la détection du coronavirus SARS-CoV-2 dans des prélèvements rectaux chez des patients ayant manifesté des signes gastro-intestinaux (diminution de l’appétit, nausée, vomissement et diarrhée). Les personnes infectées asymptomatiques pourraient aussi excréter le virus par les selles sans le savoir [78].

Par ailleurs, le SARS-CoV-2 a aussi été détecté chez des animaux. En effet, des cas sporadiques de contamination d’animaux domestiques et sauvages en captivité ont été décrits et des infections expérimentales ont permis de démontrer la réceptivité de quelques espèces animales au virus [6, 55].

Actuellement, les données disponibles sur la propagation du virus dans les milieux hydriques sont de plus en plus étudiées par les chercheurs, notamment afin de caractériser ce pathogène, comprendre son mode de contagion et standardiser les techniques permettant son dépistage.

Une des nombreuses questions pertinentes qui ont été posées et nécessitent une réponse concluante est: « Le SARS-CoV-2 risque-t-il de se propager dans la mer et les réservoirs d’eau douce *via* les réseaux d’eaux usées ? ».

Cette publication constitue une revue de littérature. Elle a pour objet d’évaluer l’importance de la contamination des milieux aquatiques par le SARS-CoV-2, ainsi que d’estimer les impacts notamment zoosanitaires dans ces milieux.

## Généralités sur les coronavirus et le SARS-COV-2

**Figure 1:**
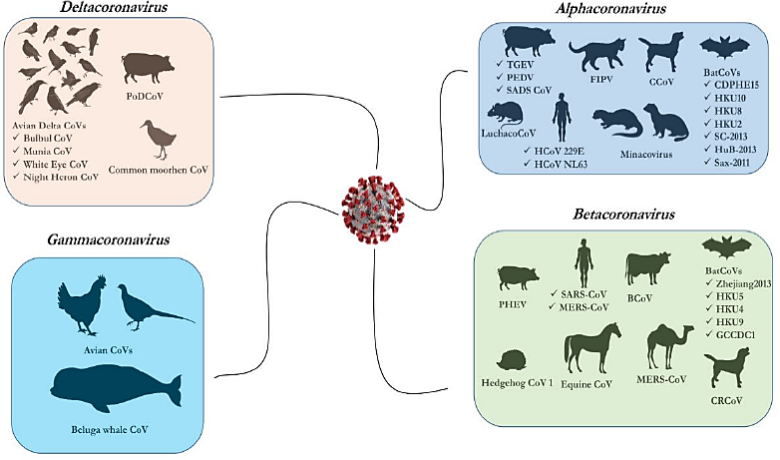
Les quatre genres de Coronavirus [49] The four different genera of Coronavirus [49]

Les coronavirus appartiennent à l’ordre des Nidovirales, famille des Coronaviridae, sous-famille des Orthocoronavirinae. Ils sont regroupés en quatre genres: *Alphacoronavirus, Betacoronavirus, Gammacoronavirus* et *Deltacoronavirus* (Fig. 1). Comme tout virus à ARN, les mutations et les recombinaisons génétiques sont fréquentes chez les coronavirus, ce qui explique leur capacité de franchissement de la barrière d’espèce [42].

Le troisième coronavirus émergent ayant frappé l’humanité au cours du XXI^e^ siècle, après le SARS-CoV-1 en 2002 et le MERS-CoV en 2012, a été nommé « SARS-CoV-2 » par le Comité international de taxonomie [66]. Ce nouveau virus appartient au genre des *Betacoronavirus,* sous-genre *Sarbecovirus.* Il est génétiquement distinct de MERS-CoV (Coronavirus du syndrome respiratoire du Moyen-Orient) et de SARS-CoV-1 (syndrome respiratoire aigu sévère) et présente, respectivement, 50 % et 79 % d’identité nucléotidique avec eux. Ces derniers virus avaient une origine animale et ont été identifiés, respectivement, en Arabie saoudite en 2012 et en Chine en 2002 [5].

L’origine exacte du SARS-CoV-2 reste une énigme à résoudre par les chercheurs. De nombreuses études scientifiques ont été conduites à cet égard, révélant qu’une source animale serait possible. D’après l’étude de Zhou et al en 2020, le virus présente 96 % d’identité nucléotidique avec celle d’un virus de chauve-souris (Bat CoV RaTG13) du genre *Rhinolophus* suggérant que l’un des ancêtres de l’agent de la COVID-19 aurait été hébergé chez une espèce de chauve-souris [11]. Des preuves concurrentes ont également fait incriminer les pangolins comme une espèce intermédiaire potentielle pour l’émergence du SARS-CoV-2. En effet, un coronavirus isolé chez des pangolins (genre *Manis)* confisqués par les services de douanes au Guangdong début 2019 présente une similitude de 99 % de son domaine RBD (domaine récepteur-grippant) de la protéine Spike avec celui de SARS-CoV-2, permettant ainsi une recombinaison avec les récepteurs ACE2 humains, mais le reste du génome est nettement plus distant [41, 45, 75].

La stabilité du SARS-CoV-2 est semblable à celle du SARS-CoV-1. En effet, une étude a montré que des virus viables dans les aérosols pouvaient être détectés jusqu’à 3 heures après l’aérosolisation, jusqu’à 4 heures sur le cuivre, et jusqu’à 24 heures sur le carton. De plus, ces deux virus présentent une viabilité relativement longue sur l’acier inoxydable et le polypropylène par rapport au cuivre ou au carton: l’estimation de la demi-vie médiane du SARS-CoV-2 est d’environ 13 heures sur l’acier et d’environ 16 heures sur le polypropylène [68].

## Sources de contaminations potentielles du SARS-COV-2 dans les milieux hydriques

**Figure 2:**
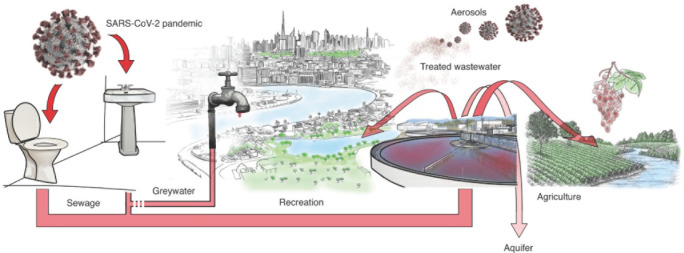
Transfert des virus entériques humains vers l’environnement et les milieux aquatiques [9] Transfer of human enteric viruses to the environment and aquatiC environment [9]

Il est admis que le SARS-CoV-2 se transmet, principalement, par voie respiratoire via l’inhalation des gouttelettes infectées (salive, expectoration). Toutefois, le virus et son ARN ont été détectés dans d’autres excrétions corporelles telles que les urines et les matières fécales déversées dans les eaux usées [36] ainsi que dans les effluents provenant des hôpitaux [69]. Ceci suggère que le risque de transmission fécale-orale d’origine hydrique du SARS-CoV-2 existe notamment dans les pays en développement utilisant souvent des eaux contaminées par les eaux usées pour l’irrigation et ayant de mauvais systèmes de traitement de l’eau [12]. La Figure 2 illustre le modèle conceptuel des voies de transfert des virus entériques humains, incluant le SARS-CoV-2, vers l’environnement et les milieux aquatiques [9].

La périphérie des écosystèmes aquatiques tels que les lacs, les rivières et les étangs constituent aussi un habitat important pour les chauves-souris, le plus grand groupe connu de réservoirs de mammifères pour les coronavirus. En déféquant dans ces endroits, ces mammifères peuvent potentiellement apporter une contribution pertinente dans le dépôt direct de matières fécales dans l’eau [61].

Étant donné que le matériel génétique de ces pathogènes entériques ne peut se retrouver dans les ressources naturelles en eau que si ces dernières sont contaminées par des eaux usées [27, 69], le suivi du SARS-CoV-2 dans les eaux a été largement déployé à travers le monde depuis le début de la pandémie. Quelques exemples d’études sont cités ci-dessous.

**Chine**: une étude menée au début du mois de mars 2020, au dernier stade de l’épidémie de COVID-19 à Wuhan, a révélé la présence de l’ARN du SARS-CoV-2 dans les déchets liquides des établissements médicaux, mais aussi dans le réseau d’égouts urbains. Cette étude a porté sur un nombre très limité d’échantillons et la trace de l’ARN du SARS-CoV-2 détectée par qPCR n’indiquait pas la présence de particules virales infectieuses [79].

**Pays-Bas**: des échantillons d’eaux usées de 6 villes et d’un aéroport ont été testés au SARS-CoV-2 lors de l’épidémie de COVID-19. Tous les échantillons ont été testés à l’aide de quatre tests qRT-PCR; trois ciblant le gène de la nucléocapside (N1-N3) et un ciblant le gène de l’enveloppe (E). Les eaux usées de l’aéroport de Schiphol à Tilburg ont été trouvées chargées en particules virales, seulement quatre jours après que le pays a détecté son premier cas de contamination humaine [53].

**France**: une analyse quantitative temporelle du SARS-CoV-2 par RT-qPCR a été réalisée dans 23 échantillons d’eaux usées non traitées et 8 échantillons d’eaux usées traitées, collectés dans 3 grandes stations d’épuration de la région parisienne. Cette étude a montré que la présence du virus dans les eaux usées fluctuait de manière similaire au schéma observé lors de l’épidémie dans la région. Cette étude a été réalisée du 5 mars au 7 avril 2020 [44].

**Équateur**: En juin 2020, le SARS-CoV-2 a été détecté dans l’eau de rivière de la ville de Quito en Équateur, pays où l’assainissement est médiocre et où des eaux usées non traitées sont directement acheminées vers les eaux naturelles [27]. Les échantillons d’eau prélevée ont fait l’objet d’analyses qRT-PCR. L’Équateur est seulement l’un des nombreux pays, en particulier ceux du Sud, qui déversent directement leurs eaux usées dans les ressources d’eau naturelles [51].

**Australie**: la présence du virus dans les eaux usées non traitées de Brisbane a été confirmée par qRT-PCR durant les mois mars et avril 2020 [2].

**États-Unis d’Amérique**: une campagne nationale de surveillance du SARS-CoV-2 dans les eaux usées de 159 comtés de 40 États américains, couvrant 13 % de la population américaine, a été menée du 18 février au 2 juin 2020. Ainsi, sur un total de 1 751 échantillons analysés par qRT-PCR, 846 échantillons étaient positifs pour l’ARN du SARS-CoV-2. Les titres viraux des eaux usées étaient cohérents avec les indicateurs de surveillance clinique de la COVID-19 [74].

**Brésil**: le SARS-CoV-2 a été détecté le 27 novembre 2019 (5,49 ± 0,02 copies du génome/l) dans les eaux usées de Santa Catalina. Les échantillonnages ultérieurs ont été positifs jusqu’au 4 mars 2020 (coïncidant avec le premier cas de COVID-19 signalé à Santa Catalina), avec une augmentation de l’ARN du SARS-CoV-2 d’un logarithme (6,68 ± 0,02 log copie du génome/l). Les échantillons d’eau prélevée ont fait l’objet d’analyses qRT-PCR. Ceci montre que le SARS-CoV-2 circulait au Brésil depuis fin novembre 2019, bien avant la notification du premier cas en Amérique (21 janvier 2020, États-Unis) [23].

**Italie**: entre octobre 2019 et février 2020, 40 échantillons d’eaux usées ont été collectés dans le cadre de projets basés sur la surveillance des eaux usées, dans 5 stations d’épuration de 3 villes et régions du nord de l’Italie (Milan/Lombardie, Turin/Piémont et Bologne/Émilie-Romagne). Les échantillons ont fait l’objet d’analyses qRT-PCR. Au total, 15 échantillons ont été confirmés positifs [39].

**Espagne**: des scientifiques espagnols de l’Université de Barcelone ont annoncé avoir trouvé des traces du SARS-CoV-2 dans un échantillon d’eaux usées de Barcelone datant du 15 janvier 2020. Les échantillons d’eau prélevée ont fait l’objet d’analyses qRT-PCR ciblant différentes parties du génome. Cette découverte indique la circulation du virus à Barcelone bien avant la détection du premier cas humain, soit le 25 février 2020 [13].

**Suède**: dès la mi-avril 2020, l’équipe de recherche du Royal Institute of Technology collecte et analyse des échantillons hebdomadaires provenant des plans de traitement des eaux usées de Stockholm. Les résultats préliminaires de ces analyses hebdomadaires sont utilisés comme un système d’alerte précoce pour signaler les infections récurrentes avant que les patients infectés puissent être testés et donc identifiés. Après avoir été concentrés, filtrés et préparés, les échantillons ont été analysés à l’aide de la technique RT-PCR pour détecter l’ARN du SARS-CoV-2 [12].

**Inde**: le matériel génétique du SARS-CoV-2 a été détecté sur des échantillons effectués entre le 8 et le 27 mai 2020 dans une station d’épuration des eaux usées de Pirana à Ahmedabad, Gujarat. Cette station reçoit les effluents de l’hôpital civil traitant les patients atteints de COVID-19. Les échantillons collectés ont été analysés à l’aide de la technique RT-PCR [38].

**Turquie**: la recherche du SARS-CoV-2 dans les eaux usées dans 7 principales stations d’épuration d’Istanbul a démarré le 7 mai 2020. Un total de 9 échantillons a été prélevé et analysé pour la présence du SARS-CoV-2 avec la méthode qRT-PCR. Le génome du SARS-CoV-2 a été détecté quantitativement dans tous les échantillons [37].

**Pakistan**: la présence de l’ARN du SARS-CoV-2 dans les eaux usées a été investiguée en utilisant le réseau de surveillance environnementale du virus de la poliomyélite déjà implémenté au Pakistan. Un total de 21 sur 78 échantillons prélevés d’eaux usées a été testé positif avec la méthode qRT-PCR pour la détection de l’ARN du SARS-CoV-2 [63].

**Émirats arabes unis**: le matériel génétique du SARS-CoV-2 a été détecté dans des échantillons d’eaux usées issues de 2 stations de pompage parmi les 9 principales stations qui ont fait l’objet d’un échantillonnage ayant eu lieu en avril et mai 2020. Ces mêmes chercheurs ont également testé plus de 2 900 échantillons d’eaux usées municipales provenant de 49 zones distinctes de Dubaï. 28,6 % ont donné des résultats positifs. En outre, 13,6 % des échantillons d’eaux usées de 198 avions commerciaux atterrissant à l’aéroport de Dubaï ont fourni un résultat positif. Ces prélèvements ont été effectués entre mai et juillet 2020 et ont été analysés avec la méthode RT-PCR [3].

**Tunisie:** des prélèvements d’eaux usées ont été effectués lors de deux périodes différentes (septembre-octobre 2020 et février-avril 2021). L’ARN du SARS-CoV-2 a été recherché par PCR en temps réel. Ainsi, durant la première période de l’étude, l’ARN viral a été détecté dans 61,11 % des échantillons analysés collectés dans la ville de Monastir avec un taux de 88,88 % pour les eaux usées brutes et 33,33 % pour les eaux usées traitées. Ensuite, pendant la deuxième période de l’étude, l’analyse quantitative des eaux usées collectées dans 7 gouvernorats a montré la présence d’ARN viral dans environ 25 % d’entre elles avec des charges d’ARN variables. L’augmentation des quantités d’ARN viral détectées dans les eaux usées s’est accompagnée d’une augmentation du nombre de patients atteints par la COVID-19 en Tunisie [35].

## Viabilité du SARS-COV-2 dans le milieu aquatique

Le SARS-CoV-2 est un virus enveloppé: il devrait donc être moins stable dans l’environnement que les virus entériques humains non enveloppés comme les Adénovirus, les Norovirus, les Rotavirus et le virus de l’hépatite A dont la transmission par l’eau est bien connue. Dans ce cadre, plusieurs chercheurs se sont intéressés à l’étude de la viabilité du SARS-CoV-2 dans divers milieux hydriques et ont constaté que son inactivation dépend de plusieurs facteurs: la température de l’eau, la disponibilité de la lumière, la teneur en matière organique, la présence de micro-organismes, le pH et la salinité, la dilution et le traitement de l’eau [1].

### Température de l’eau

La survie extracellulaire des coronavirus dans les lacs et les rivières est différente selon la situation géographique, avec une persistance potentiellement plus longue dans les zones tempérées par rapport aux zones subtropicales et tropicales [64]. Il est bien connu que les coronavirus survivent plus longtemps dans l’environnement lorsque les températures sont relativement basses et sous des conditions d’humidité relative faible. Dans ce cadre, il a été suggéré qu’un grand nombre de cas de COVID-19 sont associés à des climats froids et secs dans les régions tempérées du monde suspectant ainsi la saisonnalité observée de la propagation du virus [1]. Ainsi, comme les autres coronavirus, le SARS-CoV-2 est très sensible à la température (Tableau I), plus stable à 4 °C et plus sensible lorsque la chaleur augmente [15].

**Tableau I:** Effet de la température de l’eau sur la survie du SARS-CoV-2 [15] Effect of water temperature on SARS-CoV-2 survival [15]

Température de l’eau	Temps de survie du SARS-CoV-2
4 °C	pas d’inactivation (au 14e jour)
22 °C	14 jours
37 °C	2 jours
56 °C	30 min
70 °C	5 min

À ce jour, il n’y a pas de données sur la survie et sur le caractère infectieux du SARS-CoV-2 dans les eaux usées, bien qu’elles soient probablement similaires à celles du SARS-CoV-1. Ce dernier a été inactivé plus rapidement dans les eaux usées à 20 °C (2 jours) qu’à 4 °C (14 jours) [70]. Ainsi, une température plus élevée diminuerait le temps de survie de ces virus à ARN enveloppés.

Aussi la profondeur de l’eau, impactant la température, devrait-elle également avoir un effet sur leur survie, puisque les zones peu profondes des écosystèmes aquatiques ont tendance à avoir des températures moyennes plus élevées [34].

### Disponibilité de la lumière

Une étude expérimentale menée par Ratnesar-Shumate et al (2020) a démontré pour la première fois que les UV-B à des niveaux représentatifs de la lumière naturelle du soleil inactivent rapidement le SARS-CoV-2 sur des surfaces, en particulier le virus séché sur de l’acier inoxydable. En effet, sous des niveaux d’ensoleillement simulant le midi au solstice d’été et le midi au solstice d’hiver à une latitude de 40° nord, 90 % du SARS-CoV-2 infectieux est inactivé, respectivement, toutes les 6,8 minutes et toutes les 14,3 minutes, dans de la salive séchée sur une surface (acier inoxydable) [60].

D’autre part, Sloan et al (2020) ont démontré que le temps d’inactivation du SARS-CoV-2 semble être plus long (107 minutes dans le mucus) par rapport aux résultats précédemment cités dans les conditions d’irradiance pendant les équinoxes de printemps et d’automne (21 mars, 21 septembre), à midi à une latitude de 40° nord [65]. Toutefois, il faut tenir compte des variations saisonnières et géographiques des UV-B pouvant de ce fait influencer l’inactivation des coronavirus [25, 30].

Les UV-A pourraient inactiver le SARS-CoV-2 par analogie au SARS-CoV-1 [48]. Cependant, Luzzatto-Fegiz et al recommandent la mise en place de recherches plus poussées et axées sur l’impact des UV-A dans l’inactivation du SARS-CoV-2 [48].

### Teneur en matière organique

Des travaux de recherches chez des sujets immunodéprimés ont montré que le SARS-CoV-2 a été cultivé à partir des selles de personnes atteintes par la COVID-19 et a provoqué un effet cytopathique 4 semaines après l’inoculation, confirmant ainsi l’hypothèse que le SARS-CoV-2 éliminé dans les selles reste infectieux [19]. Bien que les charges virales dans les selles des patients atteints de la COVID-19 soient variables, l’ARN du SARS-CoV-2 peut parfois être détecté avec des concentrations comparables à celles de nombreux virus entériques (~10^8^ virus par gramme de matières fécales) [36].

Liu et al (2021) ont inoculé le virus sur des spécimens de fèces et d’urine prélevés sur trois donneurs sains, dont deux adultes et un enfant de 7 ans. Ils ont démontré que la survie du SARS-CoV-2 dans les fèces des adultes varie de 2 à 6 heures et jusqu’à 48 heures dans celles de l’enfant. Alors que dans les urines, le virus infectieux a été détecté au maximum jusqu’à 3 jours dans celles des adultes et jusqu’à 4 jours dans celles de l’enfant [46].

La composition et la charge de la matière organique présentes dans les eaux usées influent sur la survie des coronavirus. L’adsorption de particules virales par la matière organique en suspension peut constituer une entrave à la diffusion de la lumière et entraîner la formation de clusters de virus, en particulier dans les eaux présentant des niveaux élevés de matières solides en suspension [71].

Dans ce même cadre, une étude a montré que la présence de la protéine albumine de sérum de veau a prolongé sensiblement le pouvoir infectieux du SARS-CoV-2, jusqu’à 96 heures sur le plastique polystyrène, sur l’aluminium et le verre et ce sous une humidité relative de 45 à 55 % et à une température de 19 à 21 °C [58].

### Présence de micro-organismes

Dans les systèmes hydriques, il existe des populations de micro-organismes qui sont soit présentes naturellement, soit introduites par le biais de rejets anthropiques. Ces populations sont plus ou moins adaptées aux changements environnementaux [7]. Dans le milieu aquatique et les eaux usées non traitées, il existe aussi des micro-organismes antagonistes qui s’attaquent et inactivent les virus comme les enzymes bactériennes extracellulaires, les facteurs antiviraux libérés par des algues et des actinomycètes ou encore des protozoaires [21]. Il faut cependant noter que les interactions potentielles entre les micro-organismes aquatiques et les coronavirus n’ont pas encore été explorées et les extrapolations dans ce cadre doivent être effectuées avec prudence.

### Ph et salinité

Alors que le SARS-CoV peut survivre pendant 4 jours dans des échantillons de selles diarrhéiques avec un pH alcalin (pH = 9) à température ambiante [40], il a été constaté que le SARS-CoV-2 est extrêmement stable dans une large fourchette de valeurs de pH (pH 3 à 10) à température ambiante (22 °C) [15]. La présence de sel est un facteur susceptible de contribuer à une diminution de la charge virale et à son inactivation par analogie avec des virus similaires comme le coronavirus du syndrome respiratoire aigu sévère (SARS-CoV), le coronavirus de la gastro-entérite transmissible (TGEV), le coronavirus humain 229E (HCoV), le coronavirus murin (MHV), le virus de la péritonite infectieuse féline (FIPV) [4, 26]. De plus, d’après l’Institut français de recherche pour l’exploitation de la mer (IFREMER), le SARS-CoV-2 n’est pas présent dans l’eau de mer [33].

D’un autre côté, des études indiquent que la photo-inactivation des virus se produit plus rapidement dans l’eau de mer que dans l’eau douce en raison de sa salinité [8].

### Effet de dilution

La charge virale initiale du SARS-CoV-2 dans les fèces varie dans une fourchette de 5 x 10^3^ -10^7,6^ copies d’ARN/ml de fèces. Cette charge virale diminue remarquablement (baisse de la concentration de 4 à 5 fois) lorsque les fèces sont diluées dans les eaux usées municipales. Cette dilution est due à différents facteurs: débit journalier déversé dans les égouts, eaux pluviales ou eaux parasites, et pourcentage des cas positifs parmi la population [22].

### Effet du traitement de l’eau

D’après Kitajima et al, (2020) et l’OMS, les méthodes conventionnelles centralisées de traitement de l’eau qui utilisent la filtration et la désinfection devraient notablement réduire la concentration du SARS-CoV-2 [36, 56]. Dans ce cadre, en France, la charge virale dans les eaux usées traitées a été réduite 100 fois par rapport aux échantillons d’eaux usées brutes [36].

Des chercheurs ont suivi la présence du SARS-CoV-2 dans les eaux usées et ont constaté qu’après un traitement secondaire, 11 % des échantillons étaient positifs à l’ARN du SARS-CoV-2 et après des traitements tertiaires 100 % étaient négatifs [59].

Une forte inactivation dans les stations d’épuration ne peut être obtenue qu’en utilisant la désinfection (UV, chlore, dioxyde de chlore, peroxyde d’hydrogène (TGEV)) [67, 70]. D’ailleurs, l’OMS recommande une étape de désinfection lorsque les stations d’épuration existantes ne sont pas optimisées pour éliminer les virus [56].

Toutefois, l’utilisation du chlore ainsi que d’autres désinfectants constituerait une menace écologique importante pour la faune et la flore aquatiques durant la pandémie de COVID-19 [77]. Une réduction de la quantité de désinfectants peut être obtenue en mettant en œuvre des bioréacteurs à membrane avec ultrafiltration pour séparer les particules virales de SARS-CoV-2 jusqu’à une taille de 60 à 140 nm. Dans le traitement des boues, la digestion thermophile (de 50 à 60 °C) est efficace, sur la base du consensus général selon lequel les coronavirus sont très sensibles aux températures élevées. En effet, le pouvoir infectieux du SARS-CoV-2 est perdu à 56 °C pendant 90 min (température de digestion anaérobie thermophile) [22].

Malgré la détection de virus dans les selles, il n’y a aucune preuve de transmission du SARS-CoV-2 par les eaux usées, brutes ou traitées [47, 56]. Toutefois, par analogie avec des virus similaires, le risque dépendrait de la décomposition du SARS-CoV-2 dans l’environnement aquatique ainsi que de la présence de sel, facteur susceptible de contribuer à une diminution de la charge virale et à son inactivation [26].

## Espèces réceptrices

### Récepteurs ACE2

La reconnaissance des récepteurs est un facteur essentiel pour définir l’aire de répartition des hôtes et l’infection inter-espèces du virus. La spécificité de l’hôte du SARS-CoV-2 et d’autres coronavirus est déterminée par l’utilisation des enzymes de conversion de l’angiotensine II (ACE2), protéines réceptrices qui servent de point d’entrée principal dans la cellule hôte pour le SARS-CoV-2. Ces récepteurs sont exprimés sur les cellules de nombreux organes incluant le cœur, les reins, les vaisseaux sanguins, le tractus digestif, les testicules, la sphère ORL et les poumons. Dans le règne animal, ACE2 est largement exprimé et sa structure est hautement conservée [10]. L’expression ACE2 sur les lignées cellulaires est corrélée avec la susceptibilité à l’infection entraînée par le SARS-CoV-2, ce qui suggère que l’ACE2 est un récepteur majeur pour le SARS-CoV-2 [31]. Dans ce même cadre, les travaux de Damas et al portant sur la comparaison de la similitude de 25 séquences d’acides aminés de la protéine ACE2 chez 410 espèces de vertébrés (des oiseaux, des poissons, des amphibiens, des reptiles et des mammifères) avec celle des humains dans l’objectif d’évaluer les scores de liaison ACE2/SARS-CoV-2, a pu révéler que les primates sont les animaux les plus à risque de contracter le SARS-CoV-2 *via* ACE2 [18].

### Réceptivité des animaux à la COVID-19

**Figure 3:**
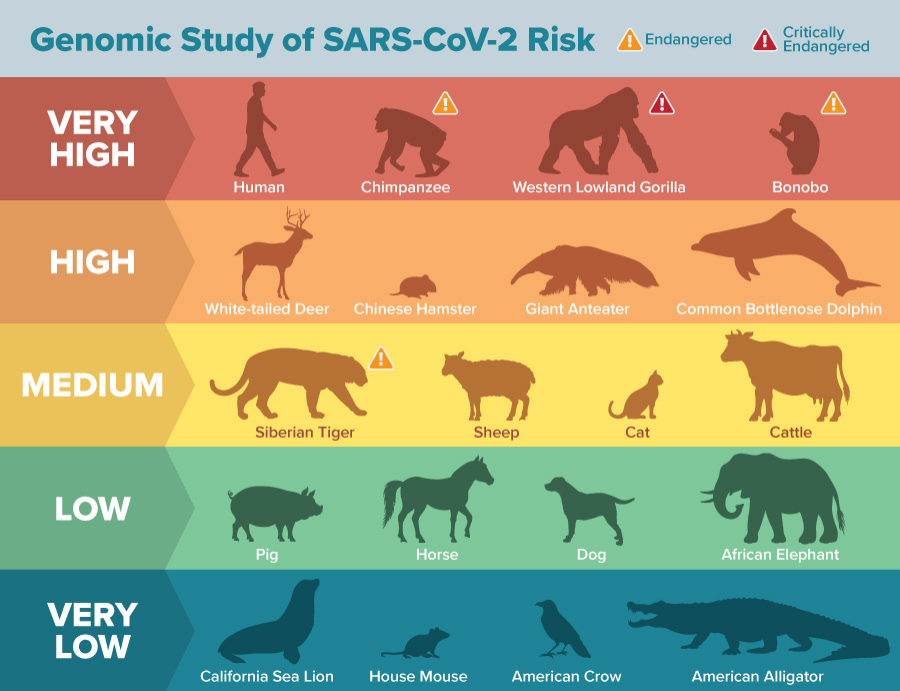
Degrés de réceptivité des espèces animales au SARS-CoV-2 [18] Animal species’ receptivity to SARS-CoV-2 [18]

La transmission du virus SARS-CoV-2 des humains à d’autres espèces animales sensibles a déjà été observée. En effet, plusieurs espèces animales en contact étroit avec des humains infectés ont été testées positives pour le SARS-CoV-2 [42], entre autres des chats, des chiens, des visons d’élevage, des félins en captivité (lion, tigre, puma, léopard des neiges), des gorilles ainsi que des loutres [54]. De nombreuses espèces de mammifères et d’oiseaux, aussi bien aquatiques que terrestres, sont touchées par des coronavirus [66]. Toutefois, la présence de coronavirus dans les organismes aquatiques n’est pas assez décrite. Dans l’objectif de comparer des séquences ACE2 de 410 espèces de vertébrés (poissons, amphibiens, oiseaux, reptiles et mammifères) et de prédire leur capacité à se lier au SARS-CoV-2, l’étude réalisée par Damas et al a pu révéler que les singes de l’Ancien Monde présentent un risque théorique d’infection très élevé (Fig. 3) [18]. En revanche, tous les monotrèmes, marsupiaux, oiseaux, poissons, amphibiens et reptiles testés ont été à risque très faible [18].

## Impacts du SARS-COV-2 sur les écosystèmes aquatiques

### Impacts directs

Les études effectuées sur des virus humains, y compris des coronavirus, n’ont pas exclu la possibilité de les détecter dans les eaux côtières puisqu’ils sont susceptibles d’être rejetés dans le milieu marin via les ruissellements urbains ou agricoles ou dans les effluents d’eaux usées [28, 70]. À cet effet, il est important de comprendre les impacts des coronavirus et en particulier du SARS-CoV-2 sur la vie aquatique.

La présence et la persistance du SARS-CoV-2 dans l’eau de mer et les eaux douces ou saumâtres n’ont pas été assez étudiées. Par comparaison à d’autres types de contaminants d’un milieu aquatique, plusieurs facteurs devraient être pris en considération pour évaluer l’impact de ces virus sur l’écosystème aquatique; en particulier, nous citons:

- la charge virale déversée en mer qui dépend de la nature de la source (eaux usées, baigneurs, débris…) et des paramètres qui ont favorisé le déversement (fortes pluies, débit du déversement.);
- les effets de la dilution qui dépend aussi de plusieurs facteurs notamment des particularités de la courantologie de la zone (embouchure des oueds et des fleuves avec la mer, le lac, la baie.);
- les caractéristiques physico-chimiques du milieu réceptif (salinité, pH, luminosité…), décrites précédemment.

Les quelques études d’impacts directs effectuées dans ce cadre ont ciblé certaines espèces de la faune aquatique, en particulier les poissons, les mollusques bivalves, les mammifères marins et les oiseaux.

### Contamination des « poissons » par le SARS-CoV-2

Depuis la pandémie de COVID-19, de nombreuses pistes ont été explorées et quelques publications scientifiques ont abordé la sensibilité des poissons vis-à-vis du SARS-CoV-2 (Tableau II). Selon les travaux de Damas et al, les classes de vertébrés autres que les mammifères ne sont pas susceptibles d’être un hôte intermédiaire ou un réservoir pour le virus, à moins que le SARS-CoV-2 utilise un autre récepteur pour l’infection [18]. Selon Bondad-Reantoso et al [10], les poissons ne possèdent pas les conditions d’hôte requises pour assurer la réplication de SARS-CoV-2 et ce pour les raisons suivantes:

- le SARS-CoV-2 appartient à la famille des Coronaviridae et au genre *Betacoronavirus,* reconnus pour infecter les mammifères et jusqu’à présent, il n’a pas été identifié de viroses de poissons transmissibles aux humains [73];
- le SARS-CoV-2 touche principalement les voies respiratoires supérieures et inférieures, en particulier les poumons. Or, la plupart des poissons à l’exception de quelques espèces n’en possèdent pas [10];
- la comparaison du récepteur ACE2 humain avec celui d’un poisson *(Callorhinchus milii)* a montré une identité de séquence d’acides aminés de l’ordre de 59 %, ce qui rend l’infection des poissons peu probable [14];
- en raison du mécanisme de thermorégulation de l’espèce, la probabilité de transmission d’une maladie infectieuse courante parmi les homéothermes est plus élevée que le taux d’infection entre animaux homéothermes et poïkilothermes [29].

Cependant, l’analyse des principaux résidus d’acides aminés de 82 poissons et de 4 espèces d’amphibiens a soutenu l’hypothèse d’une très faible affinité pour la fixation du SARS-CoV-2 par son domaine de fixation (RBD). Toutefois, une seule étude faite par Lam et al soutient l’hypothèse d’une énergie de liaison favorable à l’infection par le SARS-CoV-2 pour 10 espèces de poissons [41] (Tableau II). Ce résultat est basé sur la détermination de la différence dans les énergies libres calculées (ΔΔ*G*) qui doit être inférieure à 3,7 pour considérer que l’espèce est à risque et présente une affinité de liaison entre l’ACE2 et le domaine RBD du SARS-CoV-2 [20].

**Tableau II:** Poissons marins et d’eau douce à sensibilité prédite vis-à-vis du SARS-CoV-2 [20, 41] Marine and freshwater fish with predicted sensitivity to SARS-CoV-2 [20, 41]

Nom français	Nom scientifique	ΔΔG
Tilapia du Nil	*Oreochromis niloticus*	0,664
Daurade orientale	*Astatotilapia calliptera*	3,47
Burtoni	*Haplochromis burtoni*	3,556
Brochet	*Esox lucius*	2,123
Tambour à gros yeux	*Larimichthys crocea*	3,538
Carpe commune	*Cyprinus carpio huanghe*	2,423
Perche barramundi	*Lates calcarifer*	3,028
Brichardi	*Neolamprologus brichardi*	3,149
Citrinellum	*Amphilophus citrinellus*	2,188
Turbot	*Scophthalmus maximus*	3,218

### Contamination des mollusques bivalves et des crustacés par le SARS-CoV-2

La première (fin décembre 2019) et la deuxième vague épidémique (juin 2020) de la COVID-19 ont été respectivement associées au marché de fruits de mer à Wuhan et au marché de fruits de mer Xinfadi à Pékin, Chine, laissant suspecter le rôle des fruits de mer comme une source potentielle de transmission [17]. Toutefois, des échantillons prélevés par une équipe de l’IFREMER sur des lots de coquillages provenant de sites exposés à des sources de contamination fécale humaine, notamment 1 échantillon de palourdes, 2 échantillons de moules et 16 échantillons d’huîtres creuses prélevés en France le long des littoraux normand, breton, atlantique et méditerranéen ont permis de conclure qu’aucune trace de SARS-CoV-2 n’a été détectée dans les mollusques analysés. Cependant, l’absence de virus doit être interprétée avec précaution, car elle pourrait être la conséquence des traitements des eaux usées avant déversement en milieu marin [32].

Jusqu’à présent, aucune étude sur le potentiel des huîtres à accumuler le SARS-CoV-2, le SARS-CoV-1 ou d’autres coronavirus n’a été publiée. Un avis d’expert reçu du CEFAS *(Centre for Environment Fisheries and Aquaculture Science)* via le Defra (ministère britannique de l’environnement, de l’alimentation et des affaires rurales) affirme que les mollusques ne sont pas susceptibles d’être infectés par les bétacoronavirus et aucun récepteur ACE2 n’a été signalé chez les crustacés [16]. En outre, Zeigler Allen et al [76] ont rapporté des séquences de type coronavirus dans le plancton récupéré de la mer Baltique qui résultaient probablement d’une contamination d’origine humaine.

Selon un groupe d’éminents spécialistes dans le domaine de la santé des animaux aquatiques, de l’aquaculture, de la pêche, de la sécurité sanitaire des aliments et certains vétérinaires, ainsi que des organisations internationales telles que l’Organisation des Nations Unies pour l’alimentation et l’agriculture (FAO), il est admis qu’« à l’heure actuelle, il n’y a aucune preuve suggérant que le SARS-CoV-2 peut infecter les animaux aquatiques destinés à l’alimentation comme les poissons, les crustacés et les mollusques ». Toutefois, les animaux aquatiques et leurs produits destinés à l’alimentation, comme toute autre surface, peuvent évidemment être contaminés par le SARS-CoV-2 et devenir une source de propagation de la maladie, en particulier lorsqu’ils sont manipulés par des personnes infectées par le virus, alors que les produits cuits ne constitueraient pas un danger pour le consommateur puisque les coronavirus sont thermolabiles et ne résistent pas aux températures de cuisson normales (> 70 °C) [10].

### Contamination des mammifères marins par le SARS-CoV-2

Des coronavirus ont été isolés chez les mammifères marins, dont le béluga *(Delphinapterus leucas)* et le grand dauphin *(Tursiops aduncus)* de l’océan Indien.

Cependant, aucune information n’est disponible concernant l’écologie, la voie d’infection, le tropisme cellulaire, le mode de transmission et/ou les vecteurs putatifs des coronavirus pouvant infecter les organismes aquatiques [62].

D’une façon générale, il a été longtemps connu que seuls les genres *Alpha* et *Gammacoronavirus* touchaient des mammifères marins de l’ordre des pinnipèdes et des cétacés: phoque commun *(Phoca vitulina)* (CoV alpha et gamma), béluga *(Delphinapterus leucas)* (gamma BWCoV), grand dauphin de l’océan Indien *(Tursiops aduncus)* (gamma BdCoV) [62, 72].

En ce qui concerne le SARS-CoV-2, une équipe de l’Université Davis de Californie a révélé que 12 espèces de cétacés (baleines et dauphins) sont à risque théorique élevé d’infection [18]. Une publication plus récente utilisant une approche de modélisation a montré que 18/21 espèces de cétacés auraient même une sensibilité plus élevée que les humains [51].

**Figure 4:**
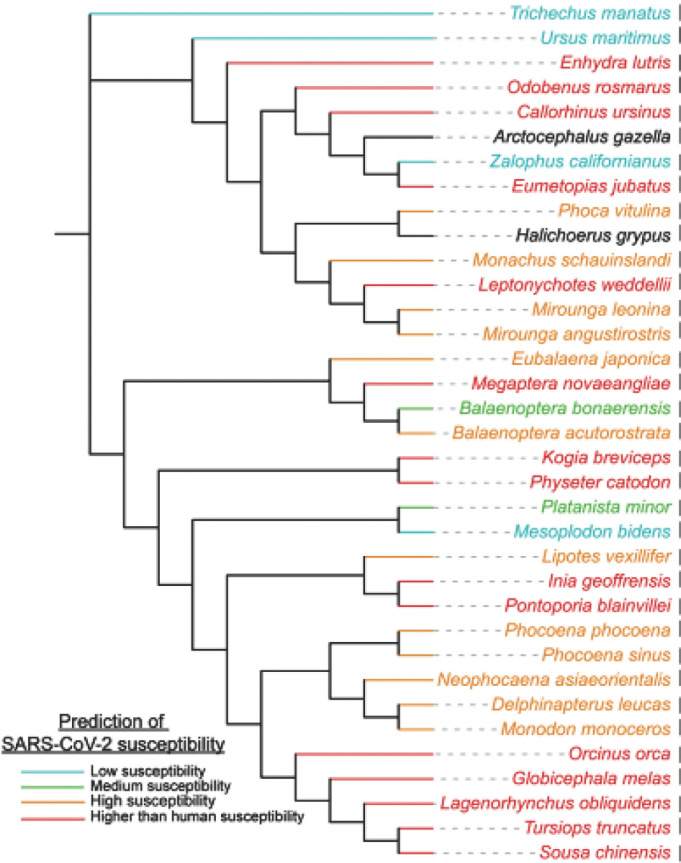
Prédiction de la sensibilité des mammifères marins vis-à-vis du SARS-CoV-2 [51] Predicted marine mammals’ sensitivity to SARS-CoV-2 [51]

Également, la majorité des espèces de pinnipèdes (phoques et otaries) (8/9) sont très sensibles au SARS-CoV-2. Parmi les Carnivores (fissipèdes), la loutre de mer *(Enhydra lutris)* est très sensible au virus, alors que les ours blancs *(Ursus maritimus)* ont une sensibilité réduite (Fig. 4).

El Masry et al [20] ont proposé une liste de mammifères marins dont la sensibilité vis-à-vis du SARS-CoV-2 pourrait être prédite à partir de différents auteurs (Tableau III).

**Tableau III:** Les mammifères marins à sensibilité prédite vis-à-vis au SARS-CoV-2 [20] Marine mammals with predicted sensitivity to SARS-CoV-2 [20]

Nom français	Nom scientifique
Marsouin aptère	*Neophocaena asiaeorientalis*
Marsouin commun	*Phocoena phocoena*
Petit rorqual de l’Antarctique	*Balaenoptera bonaerensis*
Baleine grise	*Eschrichtius robustus*
Globicéphale commun	*Globicephala melas*
Dauphin à flancs blancs du Pacifique	*Lagenorhynchus obliquidens*
Dauphin de Chine	*Lipotes vexillifer*
Orque	*Orcinus orca*
Grand dauphin	*Tursiops truncatus*
Grand cachalot	*Physeter catodon*
Phoque moine d’Hawaï	*Neomonachus (= Monachus) schauinslandi*
Baleine de Minke	*Balaenoptera acutorostrata scammoni*
Otarie de Californie	*Zalophus californianus*
Lion de mer de Steller	*Eumetopias jubatus*
Morse du Pacifique	*Odobenus rosmarus divergens*
Otarie à fourrure du Nord	*Callorhinus ursinus*
Béluga	*Delphinapterus leucas*
Baleine à bec de Sowerby	*Mesoplodon bidens*
Narval	*Monodon monoceros*

### Les oiseaux aquatiques: réservoirs de coronavirus mais faible sensibilité au SARS-CoV-2

Il est connu qu’une grande variété d’espèces d’oiseaux servait d’hôtes pour des *Gamma-*et *Deltacoronavirus.* Des recherches menées sur des oiseaux aquatiques en Australie, dans la zone de Béringie, au Brésil, au Cambodge, au Chili, à Hong Kong, en Angleterre, en Corée du Sud, en Suède, en Finlande, en Norvège et aux États-Unis ont pu identifier les espèces suivantes comme hôtes des coronavirus autres que SARS-CoV-2 (Tableau IV).

**Tableau IV:** Espèces d’oiseaux réservoirs de coronavirus [71] Bird species reservoirs of coronavirus [71]

Nom français	Nom scientifique
Sarcelle d’hiver	*Anas crecca*
Sarcelle australasienne	*Anas gracilis*
Canard colvert	*Anas platyrhynchos*
Canard à sourcils	*Anas superciliosa*
Canard pilet	*Anas acuta*
Oie rieuse	*Anser albifrons*
Oie cendrée	*Anser anser*
Oie empereur	*Anser canagicus*
Oie des neiges	*Anser caerulescens*
Oie cygnoïde	*Anser cygnoides*
Héron cendré	*Ardea cinerea*
Crabier chinois	*Ardeola bacchus*
Crabier malais	*Ardeola speciosa*
Tournepierre à collier	*Arenaria interpres*
Fuligule morillon	*Aythya fuligula*
Fuligule milouinan	*Aythya maxila*
Bernache cravant	*Branta bernicla*
Bécasseau sanderling	*Calidris alba*
Bécasseau variable	*Calidris alpina*
Bécasseau maubèche	*Calidris canutus*
Bécasseau cocorli	*Calidris ferruginea*
Bécasseau d’Alaska	*Calidris mauri*
Bécasseau semipalmé	*Calidris pusilla*
Bécasseau spatule	*Calidris pygmaea*
Bécasseau à cou roux	*Calidris ruficollis*
Guillemot colombin	*Cepphus columba*
Mouette rieuse	*Chroicocephalus ridibundus*
Harelde de Miquelon	*Clangula hyemalis*
Cygne chanteur	*Cygnuscygnus*
Dendrocygne siffleur	*Dendrocygna javanica*
Aigrette pie	*Egretta picata*
Huîtrier pie	*Haematopus ostralegus*
Goéland argenté	*Larus argentatus*
Goéland brun	*Larus fuscus*
Goéland à ailes grises	*Larus glaucescens*
Goéland bourgmestre	*Larus hyperboreus*
Goéland de la Véga	*Larusvegae*
Canard à front blanc	*Mareca americana*
Canard siffieur	*Mareca penelope*
Grand Cormoran	*Phalacrocorax carbo*
Cormoran olivâtre	*Phalacrocorax brasilianus*
Phalarope à bec large	*Phalaropus fulicarius*
Phalarope à bec étroit	*Phalaropus lobatus*
Petite Spatule	*Platalea minor*
Tadorne radjah	*Radjah radjah*
Bec-en-ciseaux noir	*Rynchops niger*
Eider à duvet	*Somateria mollissima*
Canard souchet	*Spatula clypeata*
Tadorne de Belon	*Tadorna tadorna*

Dans une enquête plus large, la prévalence des coronavirus chez les espèces d’oiseaux étudiées était très faible au Brésil (0,8 %), et supérieure à 12 % en Asie, 15 % en Australie et 19 % en Scandinavie [71]. Malgré cette particularité chez l’avifaune, deux études scientifiques ont pu détecter l’ARN d’un bétacoronavirus l’une chez des oiseaux sauvages se nourrissant de chauves-souris au Brésil, et l’autre chez une oie cendrée *(Anser anser)* échantillonnée sur un marché traditionnel en Chine [20].

La présence du virus dans les matières fécales peut contribuer au dépôt de coronavirus dans le milieu aquatique. Cela peut particulièrement concerner les espèces qui forment de grandes colonies de nidification sur les îles côtières, les fourches fluviales et dans les zones proches des lacs [71]. Toutefois la sensibilité de ces espèces d’oiseaux vis-à-vis du SARS-CoV-2 a été écartée pour les raisons suivantes [20]:

- L’analyse des principaux résidus d’acides aminés de l’ACE2 de 79 espèces d’oiseaux sauvages a démontré une faible probabilité de liaison du SARS-CoV-2; à l’exception de quelques études soutenant l’hypothèse d’une affinité de liaison probable favorisant l’infection par le SARS-CoV-2 chez certains (9 espèces) oiseaux sauvages, contredisant cependant les résultats générés par d’autres études.
- Le poulet, les canards, les oies, la dinde et la caille n’ont montré aucune sensibilité au SARS-CoV-2 après une infection expérimentale [20].

## Impacts indirects

La réduction des activités humaines durant les périodes de confinement appliqué dans de nombreux pays a certainement eu des conséquences sur les écosystèmes terrestres et aquatiques. Ainsi dans les milieux aquatiques (mer, lacs, étangs…), la baisse importante des activités de pêche, notamment celles qui utilisent des engins de pêche destructeurs sur le milieu et le stock halieutique, aurait favorisé un certain rétablissement de l’équilibre des écosystèmes. De plus, la réduction de la pollution due aux déversements des produits chimiques et des débris marins aurait certainement un impact positif sur le bien-être des animaux aquatiques. Toutefois, le rapport de l’organisation de conservation marine Oceans Asia suggère qu’en 2020 les océans ont reçu entre 4 680 et 6 240 tonnes supplémentaires de pollution plastique en raison de l’utilisation importante des masques faciaux [52].

La diminution du trafic maritime aurait également amélioré les conditions de vie des mammifères marins en réduisant les risques de collision avec les bateaux et les navires ainsi que les risques de désorientation de ces animaux engendrés par la pollution sonore. En Méditerranée, plusieurs observations ont montré que des cétacés se sont rapprochés des côtes. Ainsi, selon France Info (2020) des dauphins ont été observés dans les ports de Sardaigne, et des rorquals communs aperçus au large de la ville de Marseille [24].

En Thaïlande, plusieurs nids de tortues luths, qui sont des espèces protégées en voie de disparition, ont été découverts sur des plages où les touristes étaient interdits en raison des mesures de confinement [43].

## Conclusions

À travers cette synthèse bibliographique, il s’avère que:

- plusieurs études ont confirmé la présence du SARS-CoV-2 dans les eaux usées et les boues provenant essentiellement des excrétions (digestives et urinaires) urbaines ainsi que des déchets hospitaliers. Cette présence est un bon marqueur de la circulation virale;
- malgré la détection du virus dans les selles, il n’y a aucune preuve de transmission de la COVID-19 par les eaux usées, brutes ou traitées. Dans ce cadre, il est nécessaire de mener des recherches approfondies pour établir des méthodologies standards de suivi environnemental du SARS-CoV-2 et ainsi mieux comprendre les conséquences de sa présence dans les eaux de ruissellement, les rivières ou encore le milieu côtier;
- la destruction du SARS-CoV-2 *via* les traitements usuels des eaux dans les stations d’épuration n’est pas toujours garantie. À cet effet, il est essentiel d’élaborer un protocole de traitement efficace de ces eaux tout en veillant au respect de l’environnement;
- plusieurs études ont confirmé que la survie du SARS-CoV-2 comme d’autres coronavirus est conditionnée par les facteurs physico-chimiques comme la température, la salinité, le pH et la lumière, ce qui signifierait que la survie et la persistance de ce virus en milieu aquatique sont incertaines. Toutefois, il est fondamental d’étudier la survie du SARS-CoV-2 au niveau des embouchures des eaux de ruissellement et des rejets d’eaux usées en mer et au niveau des retenues d’eau douce;
- les études sur la sensibilité des animaux aquatiques au SARS-CoV-2 sont rares et à ce jour aucun bétacoronavirus n’a été détecté chez un animal aquatique, y compris les animaux filtreurs comme les mollusques bivalves. Toutefois, les études de prédiction basées sur la capacité de liaison du SARS-CoV-2 au récepteur ACE2 n’ont pas exclu la possibilité de contamination de certains animaux aquatiques par ce virus. Il est ainsi essentiel de poursuivre les recherches dans ce domaine pour évaluer l’implication du SARS-CoV-2 dans les écosystèmes aquatiques et la biodiversité.

## Liens d’intérêts

Les auteurs ne déclarent aucun lien d’intérêt.

## Contribution des auteurs

Tous les auteurs ont participé à la conception, rédaction, relecture et validation du manuscrit.
